# Zika: the origin and spread of a mosquito-borne virus

**DOI:** 10.2471/BLT.16.171082

**Published:** 2016-02-09

**Authors:** Mary Kay Kindhauser, Tomas Allen, Veronika Frank, Ravi Shankar Santhana, Christopher Dye

**Affiliations:** a World Health Organization, avenue Appia 20, 1211 Geneva 27, Switzerland.

## Abstract

**Objective:**

To describe the temporal and geographical distribution of Zika virus infection and associated neurological disorders, from 1947 to 1 February 2016, when Zika became a Public Health Emergency of International Concern (PHEIC).

**Methods:**

We did a literature search using the terms “Zika” and “ZIKV” in PubMed, cross-checked the findings for completeness against other published reviews and added formal notifications to WHO submitted under the International Health Regulations.

**Findings:**

From the discovery of Zika virus in Uganda in 1947 to the declaration of a PHEIC by the World Health Organization (WHO) on 1 February 2016, a total of 74 countries and territories had reported human Zika virus infections. The timeline in this paper charts the discovery of the virus (1947), its isolation from mosquitos (1948), the first human infection (1952), the initial spread of infection from Asia to a Pacific island (2007), the first known instance of sexual transmission (2008), reports of Guillain-Barré syndrome (2014) and microcephaly (2015) linked to Zika infections and the first appearance of Zika in the Americas (from 2015).

**Conclusion:**

Zika virus infection in humans appears to have changed in character as its geographical range has expanded from equatorial Africa and Asia. The change is from an endemic, mosquito-borne infection causing mild illness to one that can cause large outbreaks linked with neurological sequelae and congenital abnormalities.

## Résumé

**Objectif:**

Décrire la répartition temporelle et géographique de la maladie à virus Zika et des troubles neurologiques qui lui sont associés de 1947 au 1^er^ février 2016, date à laquelle le virus Zika est devenu une urgence de santé publique de portée internationale (USPPI).

**Méthodes:**

Nous avons réalisé une recherche bibliographique en saisissant les termes «Zika» et «ZIKV» dans PubMed, puis vérifié par recoupement l'exhaustivité des données recueillies par rapport à d'autres études publiées et ajouté à l'intention de l'OMS des avis formels soumis en vertu du Règlement sanitaire international.

**Résultats:**

De la découverte du virus Zika en Ouganda en 1947 à la déclaration d'une USPPI par l'Organisation mondiale de la Santé (OMS) le 1^er^ février 2016, 74 pays et territoires ont signalé des cas d'infections humaines par le virus Zika. La chronologie présentée dans cette étude rend compte de la découverte du virus (1947), de son isolement chez des moustiques (1948), de la première infection humaine (1952), de la propagation initiale de l'infection de l'Asie à une île du Pacifique (2007), du premier cas connu de transmission sexuelle (2008), de cas de syndrome de Guillain-Barré (2014) et de microcéphalie (2015) liés à des infections par le virus Zika et de la première apparition du virus Zika sur le continent américain (à partir de 2015).

**Conclusion:**

L’infection humaine par le virus Zika semble avoir changé de nature à mesure de l'élargissement de sa portée géographique à partir de l'Afrique équatoriale et de l'Asie. Cette infection endémique transmise par des moustiques qui provoquait des symptômes bénins est désormais à l'origine de flambées importantes associées à des séquelles neurologiques et anomalies congénitales.

## Resumen

**Objetivo:**

Describir la distribución temporal y geográfica de la infección por el virus de Zika y los trastornos neurológicos asociados, del 1947 al 1 de febrero de 2016, cuando el zika se convirtió en una emergencia de salud pública de importancia internacional (ESPII).

**Métodos:**

Se realizó un análisis documental utilizando los términos “Zika” y “ZIKV” en PubMed, se realizó una comprobación cruzada de los resultados para analizar su integridad frente a otras publicaciones y se añadieron notificaciones formales a la OMS, emitidas según el Reglamento Sanitario Internacional.

**Resultados:**

Desde el descubrimiento del virus de Zika en Uganda en 1947 hasta que la Organización Mundial de la Salud (OMS) declaró que se trataba de una ESPII el 1 de febrero de 2016, un total de 74 países y territorios registraron infecciones por el virus de Zika en humanos. El cronograma de este documento enuncia el descubrimiento del virus (1947), su aislamiento de los mosquitos (1948), el primer contagio humano (1952), la primera propagación de la infección de Asia a una isla del Pacífico (2007), el primer caso conocido de transmisión sexual (2008), informes del síndrome de Guillain-Barré (2014) y la microcefalia (2015) asociados a las infecciones de zika y la primera aparición de zika en el continente americano (a partir de 2015).

**Conclusión:**

La infección por el virus de Zika en humanos parece haber cambiado de naturaleza, conforme su alcance geográfico se ha ampliado del África ecuatorial a Asia. Este cambio se ha producido al pasar de una infección endémica transmitida por mosquitos que provocaba una enfermedad leve a una que puede derivar en grandes brotes vinculados con secuelas neurológicas y anomalías congénitas.

## ملخص

**الغرض:**

توضيح التوزيع الزمني والجغرافي لانتشار عدوى فيروس زيكا واضطرابات الجهاز العصبي المرتبطة به، منذ عام 1947 حتى 1 فبراير 2016، عندما أصبح فيروس زيكا يمثل طارئة من طوارئ الصحة العمومية التي تثير قلقًا دوليًا (PHEIC).

**الطريقة:**

أجرينا بحثًا في الكتابات باستخدام مصطلحي "Zika" و"ZIKV" في محرك البحث PubMed، وتحققنا من النتائج من حيث اكتمالها بمقارنتها بمراجعات أخرى تم نشرها وأضفنا إخطارات رسمية لمنظمة الصحة العالمية تم تقديمها بموجب اللوائح الصحية الدولية.

**النتائج:**

أبلغ عدد من الدول يبلغ في مجمله 74 دولة ومنطقة إقليمية عن وجود حالات للإصابة بعدوى فيروس زيكا، وذلك منذ اكتشاف فيروس زيكا في أوغندا عام 1974 حتى إعلان منظمة الصحة العالمية (WHO) اعتبار الإصابة بهذا الفيروس طارئة من طوارئ الصحة العمومية التي تثير قلقًا دوليًا في 1 فبراير 2016. ويمكن تسجيل مراحل اكتشاف المرض بجدول زمني في هذا التقرير على النحو التالي: اكتشاف الفيروس (1947)، وعزله من الناموس (1948)، وأول إصابة بشرية بالعدوى (1952)، والانتقال الأولي للعدوى من آسيا إلى جزر المحيط الهادئ (2007)، وأول حالة تم التعرف عليها لانتقال العدوى جنسيًا (2008)، والإبلاغ بحالات متلازمة غيّان – باريه (2014) وصغر الرأس (2015) المرتبطة بحالات الإصابة بعدوى زيكا، وأول ظهور لمرض زيكا في الأمريكتين (منذ 2015).

**الاستنتاج:**

يبدو أن الإصابة بعدوى فيروس زيكا لدى البشر تغيرت طبيعتها مع اتساع نطاق انتشارها جغرافيًا من المناطق الاستوائية في آسيا وأفريقيا. فقد ظهر التحول من العدوى المتوطنة التي ينقلها البعوض الحامل للفيروس والتي تتسبب في الإصابة بأمراض خفيفة الوطأة إلى عدوى قد تسبب حالات واسعة الانتشار ترتبط بظهور مضاعفات بالجهاز العصبي وتشوهات خلقية. ****

## 摘要

**目的:**

描述从 1947 年至 2016 年 2 月 1 日期间，寨卡病毒感染以及相关的神经系统疾病的时间和地理分布。现在，寨卡病毒已成为一项国际公共卫生紧急事件 (PHEIC)。

**方法:**

我们在 PubMed 上使用术语“寨卡”和“ZIKV”进行了文献查询，对比其他已发布文献综述交叉检查了结果的完整性，并且按照《国际卫生条例》向世界卫生组织 (WHO) 作出了有关该病毒的正式通知。

**结果:**

从寨卡病毒于 1947 年在乌干达被首次发现，到 2016 年 2 月 1 日被世界卫生组织 (WHO) 宣布为国际公共卫生紧急事件 (PHEIC)，已共有 74 个国家和地区报告出现人类寨卡病毒感染病例。该论文中的时间线跟踪记录了该病毒的发现 (1947)、从蚊子体内分离出病毒 (1948)、第一例人类感染 (1952)、从亚洲向太平洋岛屿的首次传播 (2007)、第一例已知的寨卡性传播病例 (2008)、格林-巴利综合症相关报道 (2014)、寨卡病毒感染相关的小头症 (2015) 以及美洲大陆的首例寨卡病例（自 2015 年以来）。

**结论:**

人类寨卡病毒感染的特征似乎已经改变，因为其地理范围已经开始从赤道非洲和亚洲逐渐向其他地区扩展。这是一种从引起轻微病症的地方性蚊媒感染向可引起神经系统后遗症和先天性畸形的大规模疫情的转变。

## Резюме

**Цель:**

Описать временное и территориальное распределение заражения вирусом Зика и сопряженных неврологических нарушений с 1947 года до 1 февраля 2016 года, когда вирус Зика получил статус чрезвычайной ситуации в области общественного здравоохранения, имеющей международное значение (PHEIC).

**Методы:**

Авторы выполнили поиск литературы с условиями поиска Zika и ZIKV (вирус Зика) в базе данных PubMed, подвергли результаты перекрестной сверке с результатами других опубликованных анализов, чтобы убедиться в их полноте, и добавили официальные уведомления для ВОЗ, которые были представлены в соответствии с международными медико-санитарными правилами.

**Результаты:**

С момента обнаружения вируса Зика в Уганде в 1947 году до провозглашения Всемирной организацией здравоохранения (ВОЗ) 1 февраля 2016 года чрезвычайной ситуации в области общественного здравоохранения, имеющей международное значение (PHEIC), случаи заражения человека вирусом Зика были зарегистрированы в общей сложности в 74 странах и регионах. Хронология в данном документе включает обнаружение вируса (1947 год), его выделение из комаров (1948 год), первое заражение человека (1952 год), первоначальное распространение инфекции из Азии в Океанию (2007 год), первый известный случай передачи вируса половым путем (2008 год), сообщения о синдроме Гийена-Барре (2014 год) и микроцефалии (2015 год), связанных с заражениями вирусом Зика, и первое появление вируса Зика в Америке (с 2015 года).

**Вывод:**

Судя по всему, характер заражения людей вирусом Зика изменился с расширением его географического распространения из экваториальной Африки и Азии. Эндемическая, переносимая комарами инфекция, которая вызывает болезнь, протекающую в легкой форме, трансформировалась в инфекцию, способную вызывать масштабные вспышки, связанные с неврологическими последствиями и врожденными аномалиями.

## Introduction

Zika, a flavivirus transmitted mainly by mosquitos in the genus *Aedes*, was discovered in 1947 in Uganda.^1^ From the 1960s to 1980s, human infections were found across Africa and Asia, typically accompanied by mild illness. The first large outbreak of disease caused by Zika infection was reported from the island of Yap (Federated States of Micronesia) in 2007, as the virus moved from south-east Asia across the Pacific. During an outbreak in French Polynesia in 2013–2014, Guillain-Barré syndrome was linked to Zika infection and cases of microcephaly in newborn children were also retrospectively linked to this outbreak. The World Health Organization (WHO) received the first reports of locally-transmitted infection from Brazil in May 2015. In July 2015, health ministry officials from Brazil reported an association between Zika virus infection and Guillain-Barré syndrome in adults. In October 2015, WHO received reports from Brazil of microcephaly in babies whose mothers had been exposed to Zika during pregnancy. At this time, there was no proof of a causal link between Zika infection and these neurological complications.

In February 2016, as infection moved rapidly through the range occupied by *Aedes* mosquitos in the Americas, WHO declared that Zika infection associated with microcephaly and other neurological disorders constituted a Public Health Emergency of International Concern (PHEIC). By the start of February 2016, local transmission of Zika infection had been reported from more than 20 countries and territories in the Americas and an outbreak numbering thousands of cases was under way in Cabo Verde in western Africa. Beyond the range of its mosquito vectors, Zika virus infections are expected to be carried worldwide by people as they travel and be transmitted by travellers to sexual partners who have not been to places where the virus is endemic.

## Methods

To illustrate the spread of Zika virus and associated neurological complications, we did a literature search in PubMed using “Zika” and “ZIKV” as the search terms and cross-checked our findings for completeness against other published reviews.^2^^,^^3^ In addition, we drew on formal notifications to WHO under the International Health Regulations (IHR),^4^ which are archived in the WHO Event Information Site (EIS). EIS contains information about public health events of potential international concern notified to WHO as required by the IHR. EIS notifications sometimes contain confidential patient information and therefore are not publicly available. Other details of specific events can be provided by the authors on request.

## Results

**Fig.1:**
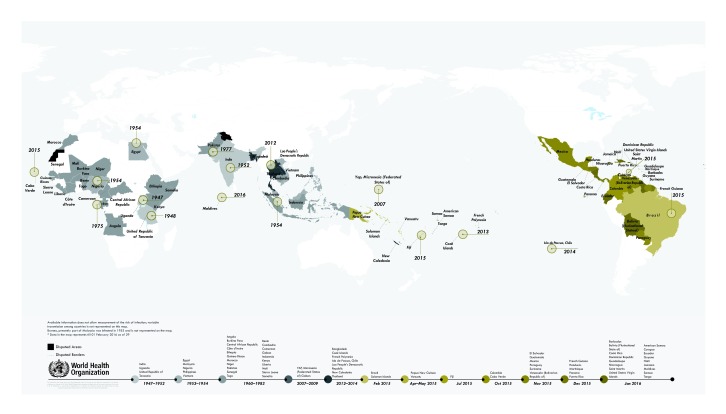
Temporal and geographical distribution of Zika virus from 1947 to February 2016

Note: Data represented up to 1 February 2016, as of 29 May 2016. Available information does not allow measurement of the risk of infection. Variable transmission among countries is not represented on this map. Infection of residents in Borneo, presently part of Malaysia, in 1953 is not represented on this map. Dates refer to events reported in the published literature^1^^,^^3^^,^^6^^–^^70^ or drawn from WHO’s Event Information Site (EIS).

The first reported case of Zika virus dates to 1947 when the virus was isolated in samples taken from a captive sentinel rhesus monkey by scientists conducting routine surveillance for yellow fever in the Zika forest of Uganda.^1^ The virus was recovered from *Aedes* (Stegomyia) *africanus*, caught on a tree platform in the forest.^1^ Laboratory infection experiments showed the virus to be neurotropic in mice.^5^ The timeline presented in this paper includes numerous serological surveys that purportedly detected antibodies to Zika virus in the 1950s and 1960s in Africa and Asia. Because serological (antibody detection) tests for Zika cross-react with antibodies stimulated by other viral infections, the presence of Zika virus is ideally confirmed by the detection of viral nucleic acids by polymerase chain reaction (PCR) testing or by virus isolation. A chronological map of the presence of Zika in those countries for which there is evidence of autochthonous transmission by mosquitos is presented in Fig. 1. The map excludes the many countries from which imported Zika infections have been reported. The country-by-country spread of Zika virus infections, from the earliest published report in 1947 to January 2014 is summarized in Table 1. Reports received by WHO in 2015 and up to 1 February 2016 are summarized in Table 2 (available at: http://www.who.int/bulletin/volumes/94/9/15-171082). 

**Table 1:** Zika virus spread and mode of transmission by country, 1947–2015

Reference	Year			Country or territory	Zika virus spread and mode of transmission	Additional information
Dick et al.^1^		1947		Uganda (Zika forest)	Virus isolated in samples taken from a rhesus monkey.	Scientists conducting routine surveillance for yellow fever in Zika forest.
Dick et al.,^1^ Smithburn^6^			1948	Uganda (Zika forest)	Virus recovered from the mosquito *Aedes* (Stegomyia) *africanus*.	The mosquito was caught on a tree platform in the Zika forest.
MacNamara et al.^7^			1951	Nigeria	Zika antibodies reported in blood samples taken from children.	Mouse protection test
Smithburn^6^			1952	Uganda	First human cases of the virus detected. First demonstration of presence of neutralizing antibodies to the virus in sera.	Neutralization test
Smithburn^6^			1952	United Republic of Tanzania (Tanganyika)	First human cases of the virus detected. First demonstration of presence of neutralizing antibodies to the virus in sera.	Neutralization test
Smithburn et al.^8^			1952	India	Confirmation of the presence of virus in humans.	Blood samples were taken from residents of Poona, India as part of a survey of immunity to the two arthropod-borne viruses, Japanese B and Russian spring-summer encephalitis. Thirty-three of 196 samples tested were shown to be reactive (neutralization test). Two of the samples neutralized only Zika virus.
Smithburn^9^			1953	British colony of Malaya	Documents the presence of neutralizing antibodies to Zika virus in sera taken from residents.	Neutralization test
Smithburn^9^			1953	British colony of North Borneo	Documents the presence of neutralizing antibodies to Zika virus in sera taken from residents.	Neutralization test
Hammon et al.^10^			1953	Philippines	Antibodies to Zika virus in blood samples from three persons.	A serological survey (neutralization test) done in the Philippines to determine types and distribution of arthropod-borne viruses in that country.
MacNamara^11^			1953	Nigeria	Zika virus infection was documented in three persons with jaundice.	Discovered during an outbreak of jaundice in eastern Nigeria. Zika virus infection was identified in two cases by a rise in serum antibodies (neutralization test) and in the one case by isolation of the virus in a 10-year-old girl. The latter is considered to be the first human Zika virus isolate.
Smithburn et al.^12^			1954	Egypt	Neutralizing antibodies to Zika virus found in one serum sample from an adult.	Neutralization test
Pond^13^			1954	Viet Nam	Antibodies to Zika virus found in sera obtained from adult residents of northern Viet Nam.	No previous reports had documented presence of the virus in this area.
MacNamara et al.^7^			1955	Nigeria	Antibodies to Zika virus in human blood samples tested.	Mouse protection test
Brès,^14^ Kokernot et al.^15^			1957	Mozambique	Antibodies to Zika virus found in sera from children and adults.	Neutralization test
Weinbren and Williams^16^			1958	Uganda (Zika forest)	Two Zika virus strains isolated from *Aedes africanus* caught in the Zika forest area.	Virus isolation
Kokernot et al.^17^			1960	Angola	A serological survey of indigenous residents showed antibodies to Zika virus.	First documented presence of Zika virus activity in the country.
Brès,^14^ Chippaux-Hyppolite^18^			1961–1962	Central African Republic	Antibodies to Zika virus found in human blood samples.	Hemagglutination assay
Sérié et al.^19^			1961–1964	Ethiopia	Antibodies to Zika virus found in human blood samples.	Samples taken in the context of yellow fever outbreak investigation.
Brès,^14^ Brès et al.^20^			1962	Senegal	Antibodies to Zika virus found in human blood samples.	Hemagglutination inhibition test
Chippaux-Hyppolite and Chippaux^21^			1963–1964	Central African Republic	Antibodies to Zika virus found in blood samples taken from four indigenous population groups.	Hemagglutination inhibition test
Brès^14^			1963–1964	Burkina Faso (Upper Volta)	Antibodies to Zika virus found in human blood samples.	Hemagglutination inhibition test
Brès,^14^ Robin et al.^22^			1963–1965	Côte d’Ivoire	Antibodies to Zika virus found in human blood samples.	Hemagglutination inhibition test
Brès,^14^ Pinto^23^			1964–1965	Guinea-Bissau (Portuguese Guinea)	Antibodies to Zika virus found in human blood samples.	Hemagglutination inhibition test
Brès^14^			1964–1966	Togo	Antibodies to Zika virus found in human blood samples.	Hemagglutination inhibition test
Brès,^14^ Salün and Brottes^24^			1964–1966	Cameroon	Antibodies to Zika virus found in human blood samples.	Hemagglutination inhibition test
Brès^14^			1964–1967	Mali	Antibodies to Zika virus found in human blood samples.	Hemagglutination inhibition test
Simpson^25^			1964	Uganda (Zika forest)	First report and confirmation that Zika virus causes human disease.	The first report from a research worker who became ill in the Zika Forest. He proved – by isolating the virus from his own blood, by infecting mice and re-isolating the virus from their blood – that Zika virus is a causative agent of human disease. The report was published with a description of the clinical features he experienced, including skin rash. Given the mild nature of his illness, the author concludes that “it is not surprising under normal circumstances the virus is not isolated frequently from man.”
Brès^14^			1965	Niger	Antibodies to Zika virus found in human blood samples.	–
Casals,^26^ Musso and Gubler,^3^ Monath et al.,^27^ Robin^28^			1965–1967	Nigeria	Antibodies to Zika virus found in human blood samples, in three separate studies.	Hemagglutination inhibition test
Brès^14^			1967	Benin (Dahomey)	Antibodies to Zika virus found in human blood samples.	Hemagglutination inhibition test
Brès^14^			1967	Gabon	Antibodies to Zika virus found in human blood samples.	Hemagglutination inhibition test
Brès^14^			1967	Liberia	Antibodies to Zika virus found in human blood samples.	Hemagglutination inhibition test
Henderson^29^			1966–1967	Uganda (north-eastern)	Antibodies to Zika virus found in human blood samples.	Hemagglutination inhibition test; from serological tests done as part of a survey of yellow fever immunity.
Henderson et al.,^29^ Geser et al.^30^			1966–1967	Kenya (north)	Antibodies to Zika virus found in human blood samples from three locations in Kenya.	Hemagglutination inhibition test; from serological tests done as part of a survey of yellow fever immunity.
Henderson et al.^29^			1966–1967	Somalia (east)	Antibodies to Zika virus found in human blood samples.	Hemagglutination inhibition test; from serological tests done as part of a survey of yellow fever immunity.
Brès^14^			1966–1967	Morocco	Antibodies to Zika virus found in people and birds.	Hemagglutination inhibition test
Henderson et al.^31^			1967–1969	Uganda	Antibodies to Zika virus found in children and adults.	Hemagglutination inhibition test
Henderson et al.^32^			1968	Kenya	Antibodies to Zika virus found in children and adults.	Hemagglutination inhibition test
Fagbami et al.^33^			1969–1972	Nigeria	Antibodies to Zika virus found in children and adults.	Neutralization test
Marchette et al.^34^			1969	Malaysia	Virus found in a pool of 29 *Aedes aegypti* mosquitoes, supporting earlier serological evidence of human infection in that area.	Virus isolated for the first time in the south-east Asia Region.
Olson et al.,^35^ Olson et al.^36^			1969–1983	Indonesia	Zika virus detected in mosquitos. Sporadic human cases occur but no outbreaks.	Seroprevalence study in Indonesia indicated widespread population exposure.
Marchette et al.^34^			1969–1983	Malaysia	Zika virus detected in mosquitos. Sporadic human cases occur but no outbreaks.	Seroprevalence study in Indonesia indicated widespread population exposure.
Darwish et al.^37^			1969–1983	Pakistan	Zika virus detected in mosquitos. Sporadic human cases occur but no outbreaks.	Seroprevalence study in Pakistan indicated widespread population exposure.
Monath et al.^27^			1970	Nigeria	Antibodies to Zika virus found in human blood samples.	Hemagglutination inhibition test
Filipe et al.^38^			1971–1972	Angola	Antibodies to Zika virus found in human blood samples.	Hemagglutination inhibition test
Renaudet et al.^39^			1972, 1975	Senegal	Antibodies to Zika virus found in human blood samples.	Hemagglutination inhibition test
Gonzales et al.^40^			1979	Central African Republic	Antibodies to Zika virus found in pygmy and non-pygmy populations.	Hemagglutination inhibition test
Adekolu-John and Fagbami^41^			1980	Nigeria	Antibodies to Zika virus found in human blood samples.	Hemagglutination inhibition test
Rodhain et al.^42^			1984	Uganda	Antibodies to Zika virus found in human blood samples.	Hemagglutination inhibition test
Monlun et al.^43^			1988, 1990	Senegal	Antibodies to Zika virus found in human blood samples.	IgM ELISA
Wolfe et al.^44^			1996–1997	Malaysia	Antibodies to Zika virus found in human blood samples.	Neutralization test
Akoua-Koffi et al.^45^			1999	Côte d’Ivoire	Antibodies to Zika virus found in human blood samples.	IgG ELISA
Filipe et al.^46^			2007	The Federated States of Micronesia (Pacific island of Yap)	First large outbreak in humans.	House-to-house surveys among the islands' small population identified suspected Zika virus disease.
Foy et al.^47^			2008	Senegal	Possibly the first documented case of sexual transmission of an infection usually transmitted by insects.	A scientist from United States of America (USA) conducting field work in Senegal falls ill with Zika infection upon his return home to Colorado and infects his wife.
Fokam et al.^48^			2010	Cameroon	Antibodies to Zika virus found in human blood samples.	Hemagglutination inhibition and complement fixation tests
Heang et al.^49^			2010–2015	Cambodia	Sporadic cases of Zika virus infection reported by travellers returning to their home country from visiting this country.	Mosquito-borne transmission of Zika virus was ongoing in the places that travellers had visited.
Kwong et al.,^50^ Perkasa et al.^51^			2010–2015	Indonesia	Sporadic cases of Zika virus infection reported by travellers returning to their home country from visiting this country.	Mosquito-borne transmission of Zika virus was ongoing in the places that travellers had visited.
Tappe et al.^52^			2010–2015	Malaysia	Sporadic cases of Zika virus infection reported by travellers returning to their home country from visiting this country.	Mosquito-borne transmission of Zika virus was ongoing in the places that travellers had visited.
Alera et al.^53^			2010–2015	Philippines	Sporadic cases of Zika virus infection reported by travellers returning to their home country from visiting this country.	Mosquito-borne transmission of Zika virus was ongoing in the places that travellers had visited.
Buathong et al.,^54^ Fonseca et al.^55^			2010–2015	Thailand	Sporadic cases of Zika virus infection reported by a Canadian returning to his home country from visiting this country.	Mosquito-borne transmission of Zika virus was ongoing in the places that travellers had visited.
Korhonen et al.^56^			2010–2015	Maldives	Sporadic cases of Zika virus infection reported by travellers returning to their home country from visiting this country.	Mosquito-borne transmission of Zika virus was ongoing in the places that travellers had visited.
Roth et al.,^57^ Cao-Lormeau and Musso,^58^ Tognarelli et al.,^59^ Dupont-Rouzeyrol et al.,^60^ Ioos et al.,^61^ Aubry et al.,^62^ Aubry et al.^63^			2011–2014	French Polynesia	The virus caused outbreaks, as in the Federated States of Micronesia in 2007.	The outbreak in French Polynesia indicated a possible association between Zika virus infection and congenital malformations and severe neurological and autoimmune complications.^61^ Serology by IgG ELISA.
Roth et al.,^57^ Cao-Lormeau and Musso,^58^ Tognarelli et al.,^59^ Dupont-Rouzeyrol et al.^60^			2013–2014	Chile (Isla de Pascua)	The virus caused outbreaks.	–
Roth et al.,^57^ Cao-Lormeau and Musso,^58^ Tognarelli,^59^ Dupont-Rouzeyrol et al.^60^			2013–2014	Cook Islands	The virus caused outbreaks.	–
Roth et al.,^57^ Cao-Lormeau and Musso,^58^ Tognarelli et al.,^59^ Dupont-Rouzeyrol et al.^60^			2013–2014	New Caledonia	The virus caused outbreaks.	–
Musso et al.^64^			2013	French Polynesia (Tahiti Island)	Zika virus isolated from patient's semen.	Additional evidence that Zika can be sexually transmitted.
Babaniyi et al.^65^			2014	Zambia	Antibodies to Zika virus found in human sera.	IgG and IgM ELISA

ELISA: enzyme-linked immunosorbent assay; Ig: immunoglobulin.

Notes: Cells with “–“ indicate no additional information.

**Table 2:** Zika virus spread and mode of transmission by country, February 2015 – January 2016

Date	Country or territory	Reported information
4 February 2015	Brazil	A ProMED-Mail posting, communicating an alert from the Caxias city government, Maranhao state, describing an outbreak of a viral disease causing fever, rash and joint pain. It was said to affect hundreds in the municipality. Report indicated chikungunya was suspected but subsequent tests were negative.^66^
2 May 2015	Brazil	From February 2015 to 29 April 2015, nearly 7000 cases of illness with skin rash were reported in Caxias city government, Maranhao state. All cases were reported to be mild, with no reported deaths. Of 425 blood samples taken for differential diagnosis, 13% were positive for dengue. Tests for chikungunya, measles, rubella, parvovirus B19 and enterovirus were negative.
15 July 2015	Brazil	Reported laboratory-confirmed Zika cases in 12 states.
17 July 2015	Brazil	Reported detection of neurological disorders associated with a history of infection, primarily from the north-eastern state of Bahia. Among these reports, 42 out of 76 (55%) were confirmed as Guillain-Barré syndrome. Among the confirmed Guillain-Barré syndrome cases, 57% (24/42) had symptoms consistent with Zika infection or dengue fever.
5 October 2015	Cabo Verde	Health centres began reporting cases of illness with skin rash, with and without fever, in the capital city of Praia, on the island of Santiago. By 14 October 2015, 165 suspected cases were reported.
8 October 2015	Brazil	Reported the results of a review of 138 clinical records of patients with a neurological syndrome, detected between March and August 2015. Of the 138 cases, 58 (42%) presented neurological syndrome with a previous history of viral infection. Of the 58 cases, 32 (55%) had symptoms consistent with Zika or dengue infection.
8 October 2015	Colombia	Reported the results of a retrospective review of clinical records which revealed the occurrence, since July 2015, of sporadic clinical cases with symptoms consistent with Zika infection. A sudden spike was reported between 11 and 26 September 2015. Altogether, 90 cases were identified with clinical symptoms consistent with, but not proven to be, Zika infection.
22 October 2015	Colombia	156 cases of Zika in 13 municipalities, with most confirmed cases concentrated in the densely populated Bolivar department.
30 October 2015	Brazil	Reported an unusual increase in the number of cases of microcephaly among newborns since August 2015, numbering 54 by 30 October.
2 November 2015	Suriname	Reported two PCR-confirmed cases of locally acquired Zika infection.
5 November 2015	Colombia	Confirmed, by PCR, 239 cases of locally acquired Zika infection.
11 November 2015	Brazil	Reported 141 suspected cases of microcephaly in Pernambuco state. Further suspected cases were being investigated in two additional states, Paraiba and Rio Grande do Norte.
12 November 2015	Suriname	Reported five PCR-confirmed cases of locally acquired Zika infection.
17 November 2015	Brazil	Reported the detection of Zika virus in amniotic fluid samples from two pregnant women from Paraiba whose fetuses were confirmed by ultrasound examinations to have microcephaly. Altogether, 399 cases of suspected microcephaly were being investigated in seven north-eastern states.
21 November 2015	Brazil	Reported that 739 cases of microcephaly are being investigated in nine states.
24 November 2015	El Salvador	Reported its first three PCR-confirmed cases of locally acquired Zika infection.
24 November 2015	French Polynesia	Reports the results of a retrospective investigation documenting an unusual increase, between March 2014 and May 2015, in the number of central nervous system malformations in fetuses and infants. At the date of reporting, at least 17 cases were identified with different severe cerebral malformations, including microcephaly and neonatal brainstem dysfunction.
25 November 2015	Mexico	Reported three PCR-confirmed cases of Zika infection, of which two were locally acquired. The third case had a travel history to Colombia.
26 November 2015	Guatemala	Reported its first PCR-confirmed case of locally acquired Zika infection.
27 November 2015	Paraguay	Reported six PCR-confirmed cases of locally acquired Zika infection. Four samples tested positive by PCR.
28 November 2015	Brazil	Reported Zika virus genome in the blood and tissue samples of a baby with microcephaly and other congenital anomalies who died within 5 minutes of birth.
28 November 2015	Brazil	Reported three deaths among two adults and a newborn associated with Zika infection. As deaths from Zika infection were extremely rare, these cases were reported in detail.
2 December 2015	Panama	Reported its first three PCR-confirmed cases of locally acquired Zika infection.
6 December 2015	Cabo Verde	Reported 4744 suspected cases of Zika. No neurological complications were reported.
14 December 2015	Panama	Reported four PCR-confirmed cases of locally acquired Zika infection and 95 cases with compatible symptoms.
15 December 2015	Cabo Verde	Additional samples taken from patients tested positive for Zika by PCR.
16 December 2015	Honduras	Reported two PCR-confirmed cases of locally acquired Zika infection.
21 December 2015	French Guiana and Martinique	Reported their first two PCR-confirmed cases of locally acquired Zika infection.
22 December 2015	Brazil	Brazilian researchers publish evidence, drawn from case reports in several countries, that depictions of Zika as “a mild cousin of dengue” may not be accurate due to the possibility of more serious disease symptoms, especially in immunocompromised patients.^71^
30 December 2015	Brazil	Reported 2975 suspected cases of microcephaly, with the highest number occurring in the north-east region.
31 December 2015	United Sates of America	Reported the first PCR-confirmed case of locally acquired Zika infection in Puerto Rico.
5 January 2016	Brazil	Researchers reported the first diagnoses of intrauterine transmission of the Zika virus in two pregnant women in Brazil whose fetuses were diagnosed, by ultrasound, with microcephaly, including severe brain abnormalities. Although tests of blood samples from both women were negative, Zika virus was detected in amniotic fluid.^72^
7 January 2016	Maldives	Reported that a Finnish national who worked in the country became ill upon his return to Finland, where he tested positive for Zika infection by PCR.
7 January 2016	Suriname	Scientists in Guyana published the results of Zika genome sequencing of viruses from four patients in Suriname whose sera were negative for dengue and chikungunya viruses but positive for Zika virus. Suriname strains belong to the Asian genotype and are almost identical to the strain that circulated in French Polynesia in 2013.^73^
7 January 2016	Brazil	Ophthalmologists in Brazil reported severe ocular malformations in three infants born with microcephaly.
12 January 2016	Brazil	In collaboration with health officials in Brazil, the US Centers for Disease Control and Prevention released laboratory findings of four microcephaly cases in Brazil of two newborns who died in the first 24 hours of life and two miscarriages, which indicated the presence of Zika virus RNA by PCR and by immunohistochemistry of brain tissue samples of the two newborns. In addition, placenta of the two fetuses miscarried during the first 12 weeks of pregnancy tested positive by PCR. Clinical and epidemiological investigations in Brazil confirmed that all four women presented fever and rash during their pregnancy. The findings were considered the strongest evidence to date of an association between Zika infection and microcephaly.
14 January 2016	Guyana	Reported its first PCR-confirmed case of locally acquired Zika infection.
15 January 2016	Ecuador	Reported its first two PCR-confirmed cases of locally acquired Zika infection. The next day, the country confirmed an additional six cases, of which two were locally acquired, three imported from Colombia and one imported from the Bolivarian Republic of Venezuela.
15 January 2016	Barbados	Reported its first three PCR-confirmed cases of locally acquired Zika infection.
15 January 2016	United States of America	The Hawaii Department of Health reported a case of microcephaly in Hawaii, born to a woman who had resided in Brazil early in her pregnancy.
16 January 2016	Bolivia (Plurinational State of)	Reported its first PCR-confirmed case of locally acquired Zika infection.
18 January 2016	Haiti	Reported its first five PCR-confirmed cases of locally acquired Zika.
18 January 2016	France (Saint Martin)	France reported the first PCR-confirmed case of locally acquired Zika in Saint Martin.
19 January 2016	El Salvador	Reported an unusual increase of Guillain-Barré syndrome. From 1 December 2015 to 6 January 2016, a total of 46 cases of the syndrome were reported, including two deaths.^67^ Of the 22 patients with a medical history, 12 (55%) presented with fever and skin rash in the seven to fifteen days before the onset of symptoms consistent with Guillain-Barré syndrome.
21 January 2016	Brazil	Reported 3893 suspected cases of microcephaly, including 49 deaths. Of these, 3381 were still under investigation. In six cases, Zika virus was detected in samples from newborns or stillbirths.
22 January 2016	Brazil	Reported that 1708 cases of Guillain-Barré syndrome have been registered by hospitals between January and November 2015. Most states reporting cases were experiencing simultaneous outbreaks of Zika, chikungunya and dengue. The potential cause of the upsurge in this syndrome couldn’t be established.
23 January 2016	Dominican Republic	Reported its first 10 PCR-confirmed cases of Zika infection, of which eight were locally acquired and two were imported from El Salvador.
25 January 2016	France (Martinique)	France reports two confirmed cases of Guillain-Barré syndrome in Martinique. Both cases required admission to an intensive care unit. One patient tested positive for Zika virus infection.
25 January 2016	United States of America	Reported the first PCR-confirmed case of locally acquired Zika infection in St Croix, one of the three main islands in the United States Virgin Islands.
27 January 2016	Nicaragua	Reported its first two PCR-confirmed cases of locally acquired Zika infection.
27 January 2016	French Polynesia	Reported retrospective data on its Zika outbreak, which coincided with a dengue outbreak. From 7 October 2013 to 6 April 2015, 8750 suspected cases of Zika were reported, with 383 PCR confirmed cases and an estimated 32 000 clinical consultations (11.5% of the total population). The outbreak ended in April 2014. During the outbreak, 42 cases of Guillain-Barré syndrome were diagnosed, representing a 20-fold increase in incidence over previous years. Though 10 of these patients required admission to an intensive care unit, none died. All 42 cases tested positive for Zika and dengue. Tests excluded other known causes of Guillain-Barré syndrome, including *Campylobacter jejuni*, cytomegalovirus, human immunodeficiency virus, Epstein-Barr and herpes simplex viruses. The investigation concluded that successive dengue and Zika virus infections might be a predisposing factor for developing Guillain-Barré syndrome.
28 January 2016	Curaçao	Reported its first PCR-confirmed case of locally acquired Zika.
29 January 2016	Suriname	Reported 1107 suspected cases of Zika, of which 308 were confirmed, by PCR, for Zika virus.
30 January 2016	Jamaica	Reported its first PCR confirmed case of locally acquired Zika.

PCR: polymerase chain reaction; RNA: ribonucleic acid; WHO: World Health Organization.

Notes: Reported to WHO as required by the International Health Regulations.^4^

Data source: WHO.^4^

## 1947 to 1959

As shown in Table 1, Zika virus was first isolated from rhesus monkeys. The first human cases were detected in Uganda and the United Republic of Tanzania in 1952 in a study demonstrating the presence of neutralizing antibodies to Zika virus in sera.^74^ During the same year, there was confirmation of the presence of the virus in humans in India.^8^ Throughout the 1950s, the presence of neutralizing antibodies against Zika virus was identified by serological surveys done in Egypt, Malaysia, Mozambique, Nigeria, the Philippines^10^ and Viet Nam.^13^

## 1960 to 1999

Between 1960 and 1999, Zika virus was being detected in mosquitos and sentinel rhesus monkeys used for field research in a narrow band of countries that stretch across equatorial Africa (Table 1). Altogether, the virus was isolated from more than 20 mosquito species, mainly in the genus *Aedes*. Sporadic human cases were identified, mostly by serological methods, but such cases were rare and the disease was regarded as benign. No deaths or hospitalizations were reported and seroprevalence studies consistently indicated widespread human exposure to the virus.^68^^,^^69^^,^^75^^–^^80^ Molecular studies of viruses later mapped the disease as it moved from Uganda to West Africa and Asia in the second half of the 20th century.^35^^,^^81^ Between 1969 and 1983, the known geographical distribution of Zika expanded to equatorial Asia, including India, Indonesia, Malaysia and Pakistan, where the virus was detected in mosquitos. As in Africa, sporadic human cases occurred but no outbreaks were detected and infections in humans continued to be regarded as rare, with mild symptoms. Seroprevalence studies in Indonesia, Malaysia and Pakistan indicated widespread population exposure.^34^^–^^37^ Researchers later suggested that the clinical similarity of Zika infection with dengue and chikungunya may be one reason why the disease was so rarely reported in Asia.^75^

## 2000 to 2009

In 2007, Zika virus spread from Africa and Asia to the Pacific island of Yap, in the Federated States of Micronesia, and caused the first large outbreak in humans. Before this event, only 16 cases of human Zika virus disease (including one experimental infection) had been documented worldwide (Table 3).^46^ House-to-house surveys done among the island’s population of 7391 people identified 185 cases of suspected Zika virus infections. The sample for the survey was 200 of 1276 total households. Of the 185 suspected cases, 49 (26%) were confirmed by PCR, or a specific neutralizing antibody response to Zika virus in the serum and 59 (32%) were classified as probable (patients with immunoglobulin M (IgM) antibody against Zika virus who had a potentially cross-reactive neutralizing antibody response). An estimated 73% (95% confidence interval: 68–77) of Yap residents older than three years were infected with Zika virus. No deaths, hospitalizations or haemorrhagic complications were reported.^82^^–^^84^

**Table 3:** Sixteen Zika virus infections reported in humans before the first outbreak on a Pacific island in 2007

Reference	Case number	Year	Location	Description
MacNamara^11^	1	1954	Nigeria	10-year-old African female with fever and headache.
Bearcroft^70^	2^a^	1956	Nigeria	Experimentally induced in a 34-year-old European male, residing in Nigeria for 4.5 months before inoculation; symptoms included headache and fever.
Simpson^25^	3	1964^b^	Uganda	28-year-old European male, residing in Uganda for 2.5 months before illness; with headache, rash and fever.
Moore et al.^68^	4–6	1968	Nigeria	Virus isolated from three febrile children, aged: 10 months, 2.5 years and 3 years; no clinical details available.
Fagbami^69^	7,8	1979^c^	Nigeria	2.5-year-old boy with fever; 10-year-old boy with fever, headache and body pains. 40% persons tested had neutralizing antibodies to Zika virus, demonstrating high prevalence of immunity in Nigeria. Unreported cases likely misdiagnosed as malaria.
Filipe et al.^46^	9^a^	1973	Portugal	Male arbovirus laboratory worker who had been vaccinated against yellow fever 2 months before infection; presented with chills, fever, sweating, retro-orbital and joint pain and cervicalgia.
Olson et al.^35^	10–16	1981^d^	Indonesia	Seven cases in hospitalized patients, males and females between the ages of 12 and 32 years. All cases had fever; none had rash.

^a^ Not naturally occurring.

^b^ Infection occurred 1962–1963; case published 1964.

^c^ Blood specimens for the two isolates were collected in July 1971 and May 1975; cases published in 1979.

^d^ Cases occurred in 1977 and 1978; published 1981.

Note: All infections were confirmed by virus isolation.

Although wind-blown mosquitoes can travel distances of several hundred kilometres over the open ocean, introduction of the virus by travel or trade involving an infected person or an inadvertently imported mosquito is considered the most likely source of this outbreak, especially as no monkeys were present on the island.^75^^,^^82^ The outbreak on Yap Island showed that Zika virus could rapidly cause more than a hundred confirmed and probable cases. In the absence of any evidence that viral mutations were the reason for this change in epidemic behaviour, other explanations should be considered. The first is a lack of population immunity. Regular exposure to infection in Africa and Asia may have prevented the large outbreaks eventually seen in Pacific Islands and in the Americas. The second possible explanation is that cases of Zika virus infection were historically misattributed – due to clinical similarities – to other pathogens, including malaria, dengue and chikungunya, and the frequent co-circulation of many infectious agents in these settings. The third possibility is underreporting, due to lack of surveillance systems, the often mild nature of the disease in adults and the many other causes of rare, but serious, neurological complications in adults and in utero.

## 2010 to 2015

Between 2010 and 2015 sporadic cases of Zika virus infection were reported from several countries in south-east Asia.^49^^-^^56^^,^^85^ These cases included an Australian traveller returning from Indonesia in 2012, a Canadian traveller returning from Thailand in 2013, two German travellers; one returning from Thailand in 2013, and one from Malaysian Borneo in 2014, and a Finnish traveller returning from the Maldives in 2015. We may infer that mosquito-borne transmission of Zika virus was ongoing in the places that these travellers had visited. The presence of infection was also reaffirmed in Africa (Cameroon).

In 2012, researchers published genetic sequences of Zika virus strains collected in Cambodia, Malaysia, Nigeria, Senegal, Thailand and Uganda, and constructed phylogenetic trees to assess the relationships between them.^75^ Two geographically-distinct lineages of the virus, African and Asian, were identified, as well as multiple strains within each lineage. Analysis of viral samples from Yap Island strengthened previous epidemiological evidence that the outbreak on Yap Island originated in south-east Asia.^54^^,^^75^^,^^82^^,^^84^ Between 2013 and 2014, the virus caused outbreaks in four other groups of Pacific islands: French Polynesia; Isla de Pascua (Chile); the Cook Islands; and New Caledonia.^57^^–^^60^ The outbreak in French Polynesia generated thousands of suspected infections and was intensely investigated. The results of retrospective investigations were reported to WHO on 24 November 2015 and 27 January 2016. These reports indicated a possible association between Zika virus infection and congenital malformations and severe neurological and autoimmune complications.^61^ In particular, an increase in the incidence of Zika infection towards the end of 2013 was followed by a rise in the incidence of Guillain-Barré syndrome.^47^^,^^86^ This finding challenged previous assumptions that Zika infection causes only mild illness in humans.^74^^,^^83^^,^^87^

During the 2013–14 outbreak of Zika virus in French Polynesia, two mothers and their newborn babies had Zika virus infection confirmed by PCR done on serum samples collected within four days of birth. Because they were able to show evidence of infection in the first week of life, the researchers concluded that Zika virus had been acquired by transplacental transmission or during delivery.^88^ During the same outbreak, 1505 asymptomatic blood donors were reported to be positive for Zika virus by PCR. These findings alerted authorities to the risk of post-transfusion Zika fever.^89^ In October 2015, Brazilian health ministry officials reported to WHO the results of a review of 138 clinical records of patients with a neurological syndrome, detected between March and August. Of the 138 cases, 58 (42%) presented neurological syndromes with a previous history of viral infection. Of the 58 cases, 32 (55%) had symptoms consistent with Zika or dengue infection.

## 2016

On 1 February 2016 the Director-General of WHO declared that the recent association of Zika infection with clusters of microcephaly and other neurological disorders constituted a PHEIC. Before this declaration, three epidemiologic alerts were issued by the Pan American Health Organization (PAHO) and WHO. The first epidemiological alert had been issued on 7 May 2015.^90^ Six months later – 17 November 2015 – PAHO and WHO had issued a second epidemiological alert asking PAHO Member States to report congenital microcephaly and other central nervous system malformations under the International Health Regulations.^91^ On 1 December 2015, PAHO and WHO had issued another alert to the association of Zika virus infection with neurological syndrome and congenital malformations in the Americas. The alert included guidelines for laboratory detection of the virus.^92^ On 15 January 2016, the United States of America issued interim travel guidance for pregnant women which, “out of an abundance of caution”, advised pregnant women in any trimester to consider postponing travel to areas with ongoing local transmission of the virus, or to take precautions against mosquito bites if they must travel.^93^

## Discussion

Results of previous studies in which Zika virus was confirmed by serological surveys need to be interpreted with some caution, as they used different serological methods with varying degrees of specificity. These methods include hemagglutination inhibition, complement fixation and enzyme-linked immunosorbent assays, in addition to more specific sero-neutralization tests. The fact that Zika virus cross-reacts immunologically with dengue, other flaviviruses and chikungunya, which frequently co-circulate with Zika and have the same vectors, complicates the interpretation of serological results. Nonetheless, the results of serological surveys, supported by the isolation of Zika virus from mosquitoes and vertebrate hosts, suggest that the virus is endemic in several African and Asian countries.^64^

Travellers who returned with Zika infections from Malaysia, the Maldives, Senegal and Thailand in 2012, were sentinels for virus circulation that may otherwise have gone undetected. Reasons why the virus may not always be detected include widespread immunological protection in the local population, the asymptomatic or mild and self-limiting nature of most Zika infections, clinical symptoms that overlap with dengue and chikungunya, the weakness of surveillance systems and the lack of specific diagnostic tests in many settings.

Therefore, additional research is needed to understand whether population immunity in endemic countries will confer protection against the epidemic strains of Zika currently circulating in Latin America and the Caribbean.

## Conclusion

Zika virus infection in humans appears to have changed in character while expanding in geographical range. The change is from an endemic arbovirus causing mild illness across equatorial Africa and Asia. From 2007 onwards, Zika virus caused large outbreaks in previously unexposed populations, and from 2013 onwards, outbreaks linked with neurological disorders including Guillain-Barré syndrome and congenital malformations, for reasons that are not yet known. The future transmission of Zika infection is likely to coincide with the global distribution of *Aedes* vectors. Person-to-person transmission, both vertically, from mother to fetus, and horizontally through sexual transmission, is also expected to continue, and we anticipate that infections will be carried widely by international travel.
